# The Role of ABC Transporters in Skin Cells Exposed to UV Radiation

**DOI:** 10.3390/ijms24010115

**Published:** 2022-12-21

**Authors:** Agnieszka Gęgotek, Elżbieta Skrzydlewska

**Affiliations:** Department of Analytical Chemistry, Medical University of Bialystok, 15-089 Białystok, Poland

**Keywords:** ABC transporters, skin cells, UV radiation, oxidative stress, MDR, TAP, CFTR, SUR, BCRP

## Abstract

ABC transporters are expressed in skin cells to protect them against harmful xenobiotics. Moreover, these transmembrane proteins have a number of additional functions that ensure skin homeostasis. This review summarizes the current knowledge about the role of specific ABC proteins in the skin, including multi-drug resistance transporters (MDR1/3), the transporter associated with antigen processing 1/2 (TAP1/2), the cystic fibrosis transmembrane conductance regulator (CFTR), sulfonylurea receptors (SUR1/2), and the breast cancer resistance protein (BCRP). Additionally, the effect of UV radiation on ABC transporters is shown. The exposure of skin cells to UV radiation often leads to increased activity of ABC transporters—as has been observed in the case of MDRs, TAPs, CFTR, and BCRP. A different effect of oxidative stress has been observed in the case of mitochondrial SURs. However, the limited data in the literature—as indicated in this article—highlights the limited number of experimental studies dealing with the role of ABC transporters in the physiology and pathophysiology of skin cells and the skin as a whole. At the same time, the importance of such knowledge in relation to the possibility of daily exposure to UV radiation and xenobiotics, used for both skin care and the treatment of its diseases, is emphasized.

## 1. Introduction

Skin, as the most external organ of the human body, is responsible for creating a physical and biochemical barrier to protect the body from harmful environmental factors. Moreover, skin also takes part in the constant interaction between the body and the environment, thus playing a pivotal role in maintaining body homeostasis [1]. For this reason, the skin has developed a number of adaptations that make it easier to perform this role. One of them is its layered structure consisting of various cell types, including keratinocytes, which are the main cells in the outermost layer, i.e., the epidermis, and fibroblasts, i.e., the basic cells of the next layer—the dermis. Despite the advanced differentiation conducted by the skin, keratinocytes and fibroblasts, as well as other less numerous cells present in the skin (melanocytes, nerve, or immune cells), are prepared for the metabolism of xenobiotics which are delivered directly from the external environment or from the bloodstream. However, the level and activity of xenobiotic-metabolizing enzymes in the skin cells are generally much lower than those in, e.g., the liver or the intestine; therefore, it is suggested that the activity of membrane transporters, located in the phospholipid structures of skin cell membranes, is responsible for the influence of exogenous substances on the functioning of the skin and the whole organism [2,3,4,5]. The best-known membrane proteins involved in this process are ABC (ATP-binding cassette) transporters, whose expression has been observed in skin cells such as keratinocytes [6], fibroblasts [7], and melanocytes [8].

## 2. ABC Transporters

**Figure 1:**
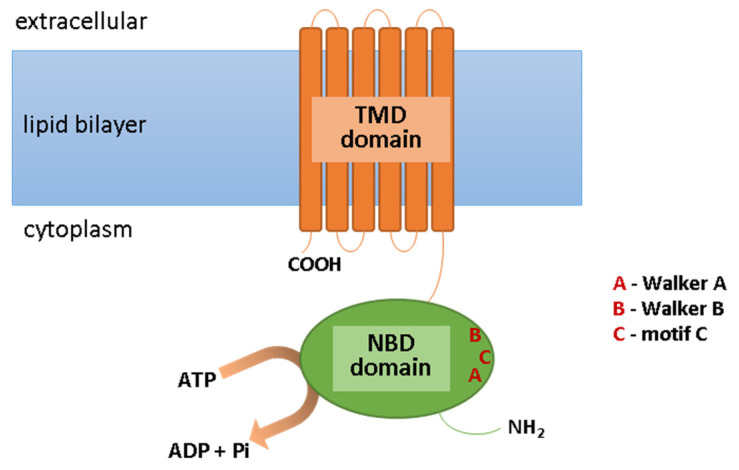
Scheme of the ABC transporter structure. Abbreviations: ADP, adenosine diphosphate; ATP, adenosine triphosphate; NBD, ATP-binding domain; Pi, phosphate; TMD, transmembrane domain.

ABC transporters are expressed in many epithelial and endothelial barrier tissues/cells, limiting the penetration of the xenobiotics between the body’s compartments. They are located, among other places, in the cells of the liver, kidneys, the epithelium of the small intestine, the blood–brain barrier, and the blood–retina barrier; they are present, however, not only in the plasma membrane, but also in intracellular membranes surrounding cell organelles, e.g., peroxisomes, lysosomes, mitochondria, and the endoplasmic reticulum [9,10,11]. They are involved in the elimination of metabolic byproducts from cells and protection against xenobiotics, including toxins, carcinogens, cytotoxic components of the diet, and drugs. ABC transporters fulfill their functions through the ejection of molecules from the cell. Usually, this process requires energy; therefore, ABC transporters have the ability to bind the ATP and to hydrolyze it to ADP and phosphate (Pi) with energy generation [12]. This is necessary for the translocation of molecules across the cell membrane, contrary to its concentration gradient, this being possible due to the specific structure of ABC transporters. These transmembrane proteins are fairly conserved in composition. Their structure includes the ATP-binding domain (NBD), which exhibits ATPase activity and is responsible for ATP hydrolysis. As a result of this reaction, the second important component of ABC transporter, i.e., the transmembrane domain (TMD), can change in conformation [13]. This is important due to the fact that TMD is the domain that recognizes substrates and marks the paths of their translocation across the cell membrane. Moreover, the motifs Walker A and Walker B are present within the NBD domain, which are characteristic of all ATP-binding proteins, as well as motif C (ABC Signature Motif), with the sequence “LSGGQ,” which is only specific for ABC proteins (Figure 1). In the construction of ABC transporters, other regions can be distinguished such as loops A, Q, D, H, and X, which affect the classification of these proteins’ subfamilies [14]. However, due to the amino acid sequence in the NBD region and its structural organization, all ABC transporters have been grouped into seven subfamilies, from ABCA to ABCG (Table 1). In addition to the systematic name, some of these transporters are known by different names, including MDR1/3 (multi-drug resistance transporter; ABCB1/4), TAP1/2 (transporter associated with antigen processing; ABCB2/3), MRP1-6 (multidrug resistance-associated protein; ABCC1-6), MRP7-9 (ABCC10-12), CFTR (cystic fibrosis transmembrane conductance regulator; ABCC7), SUR1/2 (sulfonylurea receptor; ABCC8/9), and BCRP (breast cancer resistance protein; ABCG2) [15,16].

## 3. ABC Transporters in the Skin

**Figure 2:**
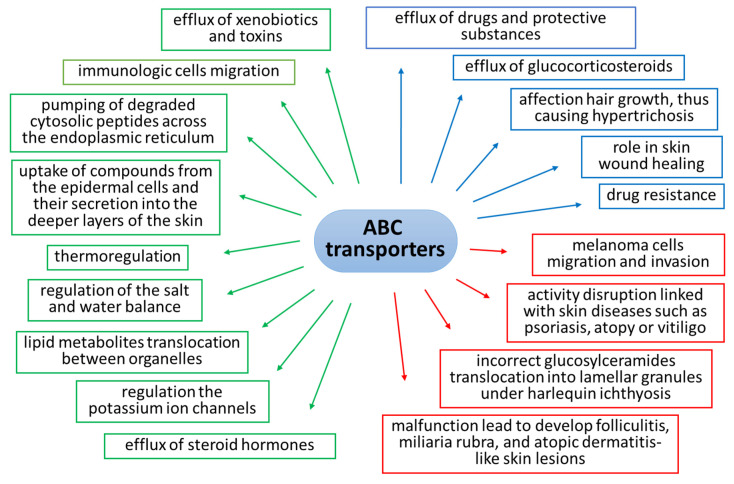
Roles and functions of ABC transporters in skin cells under physiological conditions (green), in pathological states (red), and during therapy (blue). Abbreviation: ABC, ATP-binding cassette transporter.

The expression and activity of ABC transporters in skin cells is indisputably linked to their role in skin protection against harmful xenobiotics and the oxidative stress that they induce [6]. However, the current knowledge concerning these proteins allows for the conclusion that the activity of ABC transporters is dependent on numerous factors and that they have a much wider range of action in relation to skin cells (Figure 2).

### 3.1. Activation and Suppression According to Oxidative Conditions

It is generally assumed that the appearance of an agonistic xenobiotic in the cytoplasm of the cell activates the ABC transporters and induces the efflux of this potentially harmful compound outside the cell [6]. Exposure to these compounds is very often accompanied by oxidative stress (the oxidative effect of these compounds or a side effect of their metabolism). It has not been shown that free radicals formed at the time of exposure directly affect the functioning of ABC transporters; however, there are many pathways linking oxidative stress with these transporters [17,18]. Reactive oxygen species (ROS) conjugated with GSH, glucuronide, and sulphate only are agonistic molecules for ABC transporters [19]. However, in the case of lung cancer cells, it was found that low doses of anticancer drugs, by inducing a moderate increase in ROS levels (approximately a 3-4-fold increase of the control levels), promote a defense response which results in an increase in the expression of ABC proteins, thus providing these cells with drug resistance [18]. This might be connected with an ROS-induced activity of transcription factors, such as nuclear factor-κB (NFκB), responsible for the formation of inflammation, and nuclear factor E2-related factor-2 (Nrf2), responsible for the biosynthesis of antioxidant proteins [20]. Therefore, NFκB increases the expression of ABC transporters during inflammation [21,22], while Nrf2 initiates ABC transporters in response to oxidative stress [23,24].

Oxidative stress arising from, e.g., exposure of cells/organism to pathogenic factors (exogenous and endogenous), often leads to the activation of kinases involved in intracellular signal transduction, including mitogen-activated protein kinases (MAPKs) [25]. As a result, numerous proteins are phosphorylated, including ABC transporters. The data in the literature indicate that the phosphorylation of ABC proteins is often a constitutive element of the functioning of transporters, and is necessary for their full activity, especially under oxidative conditions [26]. Moreover, MAPKs activation by ROS additionally induces NFκB and Nrf2 activity, thus favoring the expression of ABC transporters [24,27].

It is known that, while oxidative stress activates most ABC transporters, antioxidants such as vitamin C, flavonoids, or phytocannabinoids are able to suppress their activity [28,29,30,31]. Due to the recent increased public interest in aging and disease prevention, the use of herbal preparations, especially those containing high doses of natural antioxidants, has become very popular, raising the potential for interactions with the implemented drug therapies. In relation to the influence of antioxidants on ABC transporters, their action is not only based on ROS scavenging, but they are also able to inhibit drug interaction with ABC transporters during therapy, as well as prevent nucleotide hydrolysis, thus limiting the access of transporters to the energy from ATP hydrolysis [30]. Therefore, antioxidants could be considered as potential modulators of multidrug resistance and as therapeutic agents to suppress ABC transporter activity under drug-induced oxidative conditions.

### 3.2. Main Functions in the Skin

It has been reported that ABC transporters in the skin have different intensities of distribution in the epidermis compared to the dermis. For example, MRP1 has a strong expression in whole skin specimens and the dermis, and a weak expression in the epidermis [32]. This leads to the uptake of compounds from the epidermal compartment and their secretion into the deeper layers of the skin. Moreover, by coordinating the efflux of steroid hormones from normal human epidermal keratinocytes, ABC transporters ensure proper hormonal balance in the skin [33]. ABC transporters’ expression in human skin biopsies has been correlated with sweat metabolites, which indicates their role in sweat secretion and, thus, an indirect effect on body thermoregulation [34]. By removing contact allergens and exogenous compounds, such as fragments of pathogens, outside the cell, ABC transporters also play an important role in the migration of Langerhans cells and help maintain a healthy immune response in the skin [35,36]. ABC transporters also translocate lipid metabolites between cell organelles in order to regulate lipid homeostasis and prevent disease development [37]. It has been found that a dysfunction of ABCA12, which is responsible for the translocation of glucosylceramides (GlcCer) into lamellar granules, leads to a disturbance of the skin’s barrier functions and is even co-responsible for the development of a rare skin disease called harlequin ichthyosis [38].

ABC transporters also have play a significant role in melanoma, as well non-melanoma skin cancers, where the expression of these molecules is always present in a high level compared to non-cancerous human skin cells [39,40,41,42]. The exact mechanism of the ABC proteins expression in skin cancer cells is not known; however, ABC-dependent drug efflux in these cells leads to cancer multidrug resistance by decreasing intracellular drug accumulation [41,43,44,45]. Moreover, ABC transporters additionally protect the mitochondrial genome of melanoma cells against drug-induced DNA damage [43]. It has also been observed that high levels of ABC transporters in melanoma cells favors their migration and invasion, being a prognosis of numerous metastases and failure of anticancer therapy [8,46].

The presented examples are only a fragment of ABC transporters’ role in the skin that is currently known. However, it can already be seen at this stage how important they are in the functioning of cells both in normal physiology and in pathological states (Figure 2). Therefore, due to the constant exposure of skin cells to the UV radiation naturally contained in the Sun’s rays, the following question arises: what effect does UV radiation have on the expression and activity of these proteins in skin cells?

## 4. UV Radiation and ABC Transporters’ Activity

**Figure 3:**
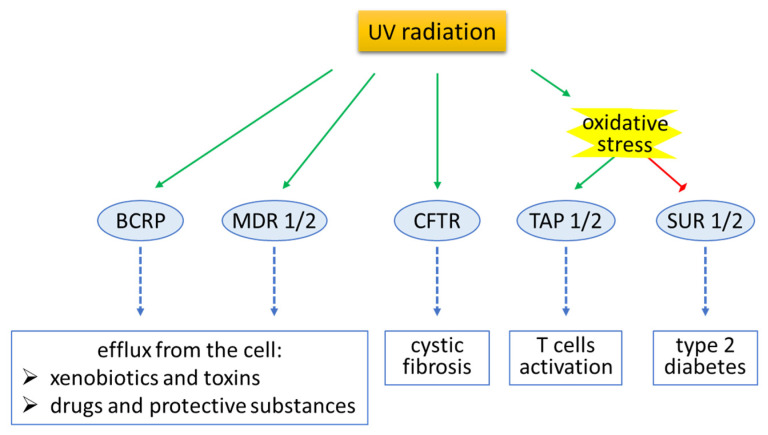
An overview of the effects of UV radiation on the basic ABC transporters in skin cells. Green arrows indicate activation; red arrows indicate suppression. Abbreviations: ABC, ATP-binding cassette transporter; BCRP, breast cancer resistance protein; CFTR, cystic fibrosis transmembrane conductance regulator; MDR, multi-drug resistance transporters; SUR, sulfonylurea receptor; TAP, transporter associated with antigen processing.

The UV radiation that reaches the surface of the Earth (UVA and UVB) is one of the most common harmful environmental factors to which cells of the human skin are daily exposed. So far it is known that UV radiation directly induces oxidative stress, disturbs the cellular lipid metabolism, leads to disorders of the structure and function of proteins, and also damages DNA molecules, thus disrupting the functioning of the exposed cells and even leading to cancer formation [47,48,49]. However, the growing public awareness of these risks means that those substances that protect or reduce the effects of UV radiation are used increasingly often [50]. For effective protection, it is often necessary for these molecules to penetrate inside the cells without their being simultaneously pumped out, e.g., by transmembrane transporters. It has been found that UV radiation (UVA and UVB) significantly increases the permeability of skin cell membranes, both through their oxidative damage and the activation of transmembrane proteins [51]. This has also been observed in the case of transporters from the ABC family [29,52]. However, some data show that UVB radiation, by impairing the generation of ATP, limits its pool in the cell, thus inhibiting the activity of ATP-dependent transporters [53]. An overview of the effects of UV radiation on the basic ABC transporters in skin cells is provided below (Figure 3).

The relationship between UV radiation and potential substrates of ABC transporters, mentioned in Table 1, is also significant for the functioning of the skin cells. It is suggested that, UV-induced protein oxidation, leading to decreased free thiol groups in peptides, may lead to a reduction in ABC transporter activity [54,55]. On the other hand, UV radiation, by decreasing cholesterol synthesis in keratinocytes [56], and increased pumping of it out from cells by the membrane transporters [57], significantly impair the structure of the cell membrane. UV radiation also has a huge impact on lipid peroxidation [58]; however, in the case of substrates for ABC transporters, they are non-oxidized compounds which are metabolized after transmembrane transport, especially in the case of peroxisomal ABC proteins [59,60].

### 4.1. Multi-Drug Resistance Transporters (MDR1/3)

The physiological functions of MDRs, especially in the skin, are poorly defined, while, under stress conditions, these are well-known transporters that mediate the efflux of chemotherapeutic agents from the intracellular space, thus inducing drug resistance [54]. It is known that the activity of MDRs in skin cells can be stimulated, e.g., by factors that induce oxidative stress [54]. Moreover, the induced activity of MDRs in the skin stimulates the migration of mononuclear phagocytes into lymphatic vessels, a process necessary for the body’s inflammatory response to a pro-inflammatory factor in the skin [36]. On the other hand, MDRs pump glucocorticosteroids out of skin cells, which is a particularly unfavorable effect during therapies for immune skin diseases, including psoriasis [55]. In the case of psoriasis, the effect of a frequent use of UV radiation as a therapeutic factor or a supporting pharmacotherapy is particularly noteworthy [56]. As reported for both health and psoriatic skin cells, the activity of MDRs during combination therapy (pharmacotherapy with phototherapy) is induced by the used therapeutic chemical, as well as UV radiation [29,52]. On the other hand, UV radiation causes an increase in the total level of oxidized proteins in cancer cells (human colon cancer cells) with enhanced expression of MDRs, compared to MDR non-stimulated cells [57]. However, these oxidative modifications do not initiate DNA repair [57]. Moreover, MDRs are sensitive to different wavelengths of UV radiation to various degrees [58]. In the case of leukemia cell line, it has been found that UVA impairs the activity of MDRs, which has not been observed in the case of UVB or UVC [58]. In all of these treatments, UV doses do not alter cell viability; hence, the authors suggest that MDRs are a physical target for oxidative damage induced directly by UVA [58]. However, the exact mechanism of the influence of UV on MBRs in skin cells is still not fully understood.

### 4.2. Transporter Associated with Antigen Processing 1/2 (TAP1/2)The physiological

TAPs, unlike the other ABC transporters, are proteins involved in the pumping of degraded cytosolic peptides across the endoplasmic reticulum into the membrane-histocompatibility complex (MHC) class I [59]. As a result, MHC displays its antigenic cargo to cytotoxic T cells on the cell surface; therefore, virus-infected or malignantly-transformed cells can be eliminated. It also induces migration and activation of immune cells (including Langerhans cells) in the skin, as well as effector functions, such as cytokine production and cytotoxicity, and may be used in epicutaneous vaccination approaches [60,61]. Hence, a disruption of the proper functioning of this transporter may lead to skin dysfunction and even disease development [62]. So far, differences in the structure of this protein have been linked to skin diseases such as psoriasis [63,64], skin atopy [65], or vitiligo [66]. Moreover, TAPs deficiency syndrome can be diagnosed based on granulomatous skin lesions before the occurrence of respiratory infectious manifestations [67,68]. Additionally, the down-regulation of TAPs in melanoma is correlated with the development of metastases and might be a marker of a poor prognosis [69].

Despite the significant role of TAPs in the skin, no clear data exist in the literature on how UV radiation affects the activity of these proteins. However, due to the fact that UV radiation induces oxidative stress, changes in TAPs activity following UV irradiation can be assumed theoretically from the data collected in the case of vitiligo [70]. In vitiligo, the described down-regulation of antioxidant enzymes, such as glutathione peroxidase 1 (GPx1), superoxide dismutase (SOD), and catalase (CAT), as well as the direct oxidizing action of UV radiation from the Sun, are the reasons for the shift in the redox balance in the oxidative direction [70]. Under conditions that simulate the effects of skin cells’ exposure to UV radiation, TAPs show a high activity, which results in T cell activation [71]. This is undoubtedly a protective reaction of the body; however, without external control, it always leads to disease symptoms.

### 4.3. Cystic Fibrosis Transmembrane Conductance Regulator (CFTR)

CFTR is a regulator of salt levels and water balance in relation to numerous body surfaces, including the skin [72]. When the protein in question is not functioning properly, chloride ions become trapped in cells [72]. The secretion of chloride ions with water and sweat outside the cells maintains the thermoregulatory function of the skin; therefore, CFTR is strongly expressed in sebaceous glands and is located on the apical side of the membrane [73]. Due to this localization, in the case of CFTR inactivation, sweat ducts become obstructed and eccrine glands become inflamed. Ultimately, this may lead to salt accumulation, resulting in folliculitis, miliary rubra, and atopic dermatitis-like skin lesions [73]. On the other hand, the up-regulation of CFTR results in Cl^-^ secretion, which has been correlated with mucous cell degranulation and the distention of the glandular ducts [74]. CFTR is also overexpressed in multiple layers of keratinocytes in the epidermis, and the protein has been found to play a significant role in skin wound healing [75]. However, the down-regulation of CFTR in in vitro-cultured human keratinocytes promotes cell migration but inhibits differentiation, while the overexpression of CFTR suppresses migration but enhances keratinocyte differentiation, indicating an important role of CFTR in the regulation of wound healing, as well as skin keratinization [75].

CFTR can be activated by many various compounds, including hormones (e.g., norepinephrine or estrogens), as well as xenobiotics (e.g., isoproterenol) [76,77]. Moreover, UV radiation belongs to the group of CFTR-activating factors; however, there are no clear data concerning the mechanism of this action. It can only be suggested that UV-induced activation of tyrosinase and tyrosinase-related proteins in melasma, or even melanoma, indirectly enhances CFTR activity [77,78]. In addition, strong exposure to UV radiation can lead to cystic fibrosis, a disease in which CFTR has been found to be overexpressed [79].

### 4.4. Sulfonylurea Receptors (SUR1/2)

SURs are transmembrane proteins that are subunits of the potassium ion channels, responsible for their opening or closing according to ATP availability. Therefore, the primary function of SURs is to sense intracellular levels of the nucleotides ATP and ADP, and monitor the energy balance within the cell [80,81]. The main molecular targets of SURs are antidiabetic drugs, the mechanism of which is action is to promote insulin release from pancreatic beta cells. High levels of glucose lead to the increased production of ATP, which, in turn, opens potassium ion channels. The resulting membrane depolarization opens voltage-dependent calcium channels, thus increasing intracellular calcium concentrations, which triggers exocytosis of insulin [82]. Data obtained in vitro suggest that SURs are also abundantly expressed in skin cells such as fibroblasts and keratinocytes [83,84]. It has been shown that chemical blocking of SUR1, as well as SUR2 action, affects hair growth, thus causing hypertrichosis [85]. Moreover, SURs have been found in the mitochondria of fibroblasts, where the regulation of potassium ion channels influences oxygen consumption, the respiratory chain, membrane potential, and the efflux of pro-apoptotic factors [86].

Due to the very small amount of data in the literature on the action of such proteins in the skin, it is even more difficult to find data on their activity under UV exposure. It can only be assumed that, after exposure to UV, these receptors behave simiarily to other cells under oxidative stress. It is known that oxidative conditions are associated with SUR suppression [87]. Therefore, it can be suggested that a malfunction of these receptors may lead to the development of type 2 diabetes, a disease additionally accompanied by oxidative stress [88]. However, that SUR reactions which are identical to oxidative stress may be occurring in the skin is only a supposition. There are also reports, however, that increased production of ROS induces different potassium channel responses (open or close), depending on the tissue [89].

### 4.5. Breast Cancer Resistance Protein (BCRP)

Another extremely important transporter protein from the ABC family is BCRP. The name itself, however, may be misleading as far as its location and function are concerned. BCRP is a transmembrane transporter of xenobiotics; however, only some of them are chemotherapeutic agents, e.g., mitoxantrone and camptothecin analogues [90]. BCRP is expressed not only in breast cancer cells, but also in the gut, the bile canaliculi, the placenta, the skin, and the blood–testis and blood–brain barriers [29,91]. Toxins and xenobiotics pumped out by these molecules limit the absorption of potentially toxic substances in cells, thus contributing to the natural resistance and longevity of normal health cells. However, malignant tissues can exploit the properties of BCRP to survive hypoxia and evade exposure to chemotherapeutic drugs [91]. In the skin, in addition to its primary role in protecting against toxins, BCRP also significantly stimulates the differentiation of activation of immune cells in response to harmful environmental factors [92]. Moreover, the action of this protein action is UV-sensitive, being activated by it [93]. Additionally, the obtained data show that UV irradiation does not cause phototoxicity nor, surprisingly, hepatotoxicity in BCRP-knockdown animals, which indicates the crucial role of this transporter not only in the skin, but also in the whole organism [93]. On the other hand, UV-induced activity of BCRP reduces the protective effect of the applied therapeutic compounds; those with antioxidant properties as well, have been observed in human keratinocytes treated with cannabidiol [29]. A similar effect has been observed in cannabidiol-treated keratinocytes isolated from psoriatic patients, which, as in the case of cancer, interferes with therapy [52].

## 5. Conclusions

The present review shows the importance of ABC transporters for the proper functioning of the human body—individual cells/tissues/organs as well as the whole organism—and the dangers to human health posed by improper control of the activity of these transporters. Exposure of skin cells to UV radiation, or the related oxidative stress, often leads to increased activity of ABC transporters, as has been observed in the case of MDRs, TAPs, CFTR, and BCRP. This is not only conducive to the pumping out of toxins, but also of protective compounds, and even drugs. A different effect of oxidative stress has been observed in the case of mitochondrial SURs, with the regulation of these channels influencing oxygen consumption, respiratory chain reactions, membrane potential, and the efflux of pro-apoptotic factors into cytoplasm. However, the limited amount of knowledge cited in this paper highlights the lack of sufficient experimental studies of ABC transporters in skin cells that would make it possible to formulate unambiguous hypotheses. At the same time, the authors show the potential importance of this knowledge in relation to healthy skin exposed to solar radiation, as well as in relation to the pharmacotherapy of various skin diseases.

## Figures and Tables

**Table 1:** The list of ABC transporters with their main functions. Abbreviations: ABC, ATP-binding cassette transporter; BCRP, breast cancer resistance protein; CFTR, cystic fibrosis transmembrane conductance regulator; MDR, multi-drug resistance transporter; MRP, multidrug resistance-associated protein; SUR, sulfonylurea receptor; TAP, transporter associated with antigen processing.

Subfamily	Transporters	Main Function
ABCA	ABCA 1-9, 12	transport of cholesterol and lipids
ABCB	ABCB 1 (MDR1), ABCB 2-3 (TAP1-2), ABCB 4 (MDR3), ABCB 5-11	transport of peptides and metabolites
ABCC	ABCC 1-6 (MRP1-6), ABCC 7 (CFTR), ABCC 8-9 (SUR1-2), ABCC 10-12 (MRP7-9)	transport of ions, cell-surface receptors
ABCD	ABCD 1-4	participate in peroxisome activation
ABCE	ABCE 1	multidrug resistance
ABCF	ABCF 1-3	regulation of innate immune response
ABCG	ABCG 1, ABCG 2 (BCRP), ABCG 4,5,8	transport of drugs, toxins, lipids, cholesterol and other steroids

## Data Availability

Not applicable.

## References

[1] Elias P.M., Wakefield J.S. (2011). Skin barrier function. Nutrition for Healthy Skin: Strategies for Clinical and Cosmetic Practice.

[2] Sleeman M.A., Watson J.D., Murison J.G. (2000). Neonatal murine epidermal cells express a functional multidrug-resistant pump. J. Invest. Dermatol..

[3] Potts R. (1997). Skin Barrier: Principles of Percutaneous Absorption. Arch. Dermatol..

[4] Prausnitz M.R., Mitragotri S., Langer R. (2004). Current status and future potential of transdermal drug delivery. Nat. Rev. Drug Discov..

[5] Takenaka S., Itoh T., Fujiwara R. (2013). Expression pattern of human ATP-binding cassette transporters in skin. Pharmacol. Res. Perspect..

[6] Osman-Ponchet H., Boulai A., Kouidhi M., Sevin K., Alriquet M., Gaborit A., Bertino B., Comby P., Ruty B. (2014). Characterization of ABC transporters in human skin. Drug Metabol. Drug Interact..

[7] Hendig D., Langmann T., Kocken S., Zarbock R., Szliska C., Schmitz G., Kleesiek K., Götting C. (2008). Gene expression profiling of ABC transporters in dermal fibroblasts of pseudoxanthoma elasticum patients identifies new candidates involved in PXE pathogenesis. Lab. Investig..

[8] Colone M., Calcabrini A., Toccacieli L., Bozzuto G., Stringaro A., Gentile M., Cianfriglia M., Ciervo A., Caraglia M., Budillon A. (2008). The multidrug transporter P-glycoprotein: A mediator of melanoma invasion?. J. Invest. Dermatol..

[9] Oldham M.L., Davidson A.L., Chen J. (2008). Structural insights into ABC transporter mechanism. Curr. Opin. Struct. Biol..

[10] Kobuchi H., Moriya K., Ogino T., Fujita H., Inoue K., Shuin T., Yasuda T., Utsumi K., Utsumi T. (2012). Mitochondrial Localization of ABC Transporter ABCG2 and Its Function in 5-Aminolevulinic Acid-Mediated Protoporphyrin IX Accumulation. PLoS ONE.

[11] Mahringer A., Fricker G. (2016). ABC transporters at the blood-brain barrier. Expert Opin. Drug Metab. Toxicol..

[12] Wilkens S. (2015). Structure and mechanism of ABC transporters. F1000Prime Rep..

[13] Jones P.M., George A.M. (2002). Mechanism of ABC transporters: A molecular dynamics simulation of a well characterized nucleotide-binding subunit. Proc. Natl. Acad. Sci. USA.

[14] Beis K. (2015). Structural basis for the mechanism of ABC transporters. Biochem. Soc. Trans..

[15] Dean M., Moitra K., Allikmets R. (2022). The human ATP-binding cassette (ABC) transporter superfamily. Hum. Mutat..

[16] Bates S.E., Robey R., Knutsen T., Honjo Y., Litman T., Dean M. (2000). New ABC transporters in multi-drug resistance. Expert Opin. Ther. Targets.

[17] Grewal G.K., Kukal S., Kanojia N., Saso L., Kukreti S., Kukreti R. (2017). Effect of oxidative stress on ABC transporters: Contribution to epilepsy pharmacoresistance. Molecules.

[18] Yuan T., Hu J., Zhu X., Yin H., Yin J. (2022). Oxidative stress-mediated up-regulation of ABC transporters in lung cancer cells. J. Biochem. Mol. Toxicol..

[19] Järvinen E., Deng F., Kiander W., Sinokki A., Kidron H., Sjöstedt N. (2022). The Role of Uptake and Efflux Transporters in the Disposition of Glucuronide and Sulfate Conjugates. Front. Pharmacol..

[20] Rubio V., García-Pérez A.I., Herráez A., Diez J.C. (2018). Different roles of Nrf2 and NFKB in the antioxidant imbalance produced by esculetin or quercetin on NB4 leukemia cells. Chem. Biol. Interact..

[21] Miller D.S. (2015). Regulation of ABC Transporters Blood-Brain Barrier. The Good, the Bad, and the Ugly. Advances in Cancer Research.

[22] Di Q., Yu N., Liu H., Hu Y., Jiang Y., Yan Y.K., Zhang Y.F., Zhang Y.D. (2011). Nuclear factor-kappa B activity regulates brain expression of P-glycoprotein in the kainic acid-induced seizure rats. Mediators Inflamm..

[23] Wang X., Campos C.R., Peart J.C., Smith L.K., Boni J.L., Cannon R.E., Miller D.S. (2014). Nrf2 upregulates ATP binding cassette transporter expression and activity at the blood-brain and blood-spinal cord barriers. J. Neurosci..

[24] Maher J.M., Dieter M.Z., Aleksunes L.M., Slitt A.L., Guo G., Tanaka Y., Scheffer G.L., Chan J.Y., Manautou J.E., Chen Y. (2007). Oxidative and electrophilic stress induces multidrug resistance-associated protein transporters via the nuclear factor-E2-related factor-2 transcriptional pathway. Hepatology.

[25] Son Y., Cheong Y.-K., Kim N.-H., Chung H.-T., Kang D.G., Pae H.-O. (2011). Mitogen-Activated Protein Kinases and Reactive Oxygen Species: How Can ROS Activate MAPK Pathways?. J. Signal Transduct..

[26] Stolarczyk E.I., Reiling C.J., Paumi C.M. (2011). Regulation of ABC Transporter Function via Phosphorylation by Protein Kinases. Curr. Pharm. Biotechnol..

[27] Li L., Cataisson C., Flowers B., Fraser E., Sanchez V., Day C.P., Yuspa S.H. (2019). Topical Application of a Dual ABC Transporter Substrate and NF-κB Inhibitor Blocks Multiple Sources of Cutaneous Inflammation in Mouse Skin. J. Invest. Dermatol..

[28] Zecchinati F., Barranco M.M., Arana M.R., Tocchetti G.N., Domínguez C.J., Perdomo V.G., Ruiz M.L., Mottino A.D., García F., Villanueva S.S.M. (2019). Reversion of down-regulation of intestinal multidrug resistance-associated protein 2 in fructose-fed rats by geraniol and vitamin C: Potential role of inflammatory response and oxidative stress. J. Nutr. Biochem..

[29] Atalay S., Dobrzyńska I., Gęgotek A., Skrzydlewska E. (2020). Cannabidiol protects keratinocyte cell membranes following exposure to UVB and hydrogen peroxide. Redox Biol..

[30] Di Pietro A., Conseil G., Pérez-Victoria J.M., Dayan G., Baubichon-Cortay H., Trompier D., Steinfels E., Jault J.M., De Wet H., Maitrejean M. (2002). Modulation by flavonoids of cell multidrug resistance mediated by P-glycoprotein and related ABC transporters. Cell. Mol. Life Sci..

[31] Morris M.E., Zhang S. (2006). Flavonoid-drug interactions: Effects of flavonoids on ABC transporters. Life Sci..

[32] Skazik C., Wenzel J., Marquardt Y., Kim A., Merk H.F., Bickers D.R., Baron J.M. (2011). P-Glycoprotein (ABCB1) expression in human skin is mainly restricted to dermal components. Exp. Dermatol..

[33] Heise R., Skazik C., Rodriguez F., Stanzel S., Marquardt Y., Joussen S., Wendel A.F., Wosnitza M., Merk H.F., Baron J.M. (2010). Active transport of contact allergens and steroid hormones in epidermal keratinocytes is mediated by multidrug resistance related proteins. J. Invest. Dermatol..

[34] Nielsen M.M.K., Aryal E., Safari E., Mojsoska B., Jenssen H., Prabhala B.K. (2021). Current state of slc and abc transporters in the skin and their relation to sweat metabolites and skin diseases. Proteomes.

[35] van de Ven R., de Jong M.C., Reurs A.W., Schoonderwoerd A.J.N., Jansen G., Hooijberg J.H., Scheffer G.L., de Gruijl T.D., Scheper R.J. (2006). Dendritic Cells Require Multidrug Resistance Protein 1 (ABCC1) Transporter Activity for Differentiation. J. Immunol..

[36] Randolph G.J., Beaulieu S., Pope M., Sugawara I., Hoffman L., Steinman R.M., Muller W.A. (1998). A physiologic function for p-glycoprotein (MDR-1) during the migration of dendritic cells from skin via afferent lymphatic vessels. Proc. Natl. Acad. Sci. USA.

[37] Tarling E.J., Vallim T.Q.d.A., Edwards P.A. (2013). Role of ABC transporters in lipid transport and human disease. Trends Endocrinol. Metab..

[38] Scott C.A., Rajpopat S., Di W.L. (2013). Harlequin ichthyosis: ABCA12 mutations underlie defective lipid transport, reduced protease regulation and skin-barrier dysfunction. Cell Tissue Res..

[39] Heimerl S., Bosserhoff A.K., Langmann T., Ecker J., Schmitz G. (2007). Mapping ATP-binding cassette transporter gene expression profiles in melanocytes and melanoma cells. Melanoma Res..

[40] Keshet G.I., Goldstein I., Itzhaki O., Cesarkas K., Shenhav L., Yakirevitch A., Treves A.J., Schachter J., Amariglio N., Rechavi G. (2008). MDR1 expression identifies human melanoma stem cells. Biochem. Biophys. Res. Commun..

[41] Fukunaga-Kalabis M., Herlyn M. (2012). Beyond ABC: Another mechanism of drug resistance in melanoma side population. J. Invest. Dermatol..

[42] Thinnes F.P. (2013). Nonmelanoma skin cancer is associated with reduced alzheimer disease risk. Neurology.

[43] Elliott A.M., Al-Hajj M.A. (2009). ABCB8 mediates doxorubicin resistance in melanoma cells by protecting the mitochondrial genome. Mol. Cancer Res..

[44] Chen K.G., Valencia J.C., Gillet J.-P., Hearing V.J., Gottesman M.M. (2009). Involvement of ABC transporters in melanogenesis and the development of multidrug resistance of melanoma. Pigment Cell Melanoma Res..

[45] Kalal B.S., Upadhya D., Pai V.R. (2017). Chemotherapy resistance mechanisms in advanced skin cancer. Oncol. Rev..

[46] Setia N., Abbas O., Sousa Y., Garb J.L., Mahalingam M. (2012). Profiling of ABC transporters ABCB5, ABCF2 and nestin-positive stem cells in nevi, in situ and invasive melanoma. Mod. Pathol..

[47] Gęgotek A., Rybałtowska-Kawałko P., Skrzydlewska E. (2017). Rutin as a Mediator of Lipid Metabolism and Cellular Signaling Pathways Interactions in Fibroblasts Altered by UVA and UVB Radiation. Oxid. Med. Cell. Longev..

[48] Gegotek A., Biernacki M., Ambrozewicz E., Surazyński A., Wroński A., Skrzydlewska E. (2016). The cross-talk between electrophiles, antioxidant defence and the endocannabinoid system in fibroblasts and keratinocytes after UVA and UVB irradiation. J. Dermatol. Sci..

[49] de Gruijl F.R. (2002). Photocarcinogenesis: UVA vs. UVB Radiation. Skin Pharmacol. Physiol..

[50] Fivenson D., Sabzevari N., Qiblawi S., Blitz J., Norton B.B., Norton S.A. (2021). Sunscreens: UV filters to protect us: Part 2-Increasing awareness of UV filters and their potential toxicities to us and our environment. Int. J. Women’s Dermatol..

[51] Gęgotek A., Bielawska K., Biernacki M., Dobrzyńska I., Skrzydlewska E. (2017). Time-dependent effect of rutin on skin fibroblasts membrane disruption following UV radiation. Redox Biol..

[52] Jarocka-Karpowicz I., Biernacki M., Wroński A., Gęgotek A., Skrzydlewska E. (2020). Cannabidiol Effects on Phospholipid Metabolism in Keratinocytes from Patients with Psoriasis Vulgaris. Biomolecules.

[53] Dumitriu I.E., Voll R.E., Kolowos W., Gaipl U.S., Heyder P., Kalden J.R., Herrmann M. (2004). UV irradiation inhibits ABC transporters via generation of ADP-ribose by concerted action of poly(ADP-ribose) polymerase-1 and glycohydrolase. Cell Death Differ..

[54] Baron J.M., Höller D., Schiffer R., Frankenberg S., Neis M., Merk H.F., Jugert F.K. (2001). Expression of multiple cytochrome P450 enzymes and multidrug resistance-associated transport proteins in human skin keratinocytes. J. Invest. Dermatol..

[55] Abe Y., Shimizu K., Katayama I. (2001). Significance of MDR1-Gene and P-Glycoprotein (P-gp) Expressions in the Lesional Skin of Psoriasis Vulgaris. Acta. Med. Nagasaki.

[56] Kemény L., Varga E., Novak Z. (2019). Advances in phototherapy for psoriasis and atopic dermatitis. Expert Rev. Clin. Immunol..

[57] Chao C.C.K., Sun N.K. (1993). Overexpression of a UV-damage recognition protein in a UV-sensitive human colon cancer cell line that features multidrug-resistant phenotype. Biochem. Biophys. Res. Commun..

[58] Trindade G.S., Capella M.A.M., Capella L.S., Affonso-Mitidieri O.R., Rumjanek V.M. (1999). Differences in Sensitivity to UVC, UVB and UVA Radiation of a Multidrug-Resistant Cell Line Overexpressing P-Glycoprotein. Photochem. Photobiol..

[59] Abele R., Tampé R. (2004). The ABCs of immunology: Structure and function of TAP, the transporter associated with antigen processing. Physiology.

[60] Stoitzner P., Tripp C.H., Eberhart A., Price K.M., Jung J.Y., Bursch L., Ronchese F., Romani N. (2006). Langerhans cells cross-present antigen derived from skin. Proc. Natl. Acad. Sci. USA.

[61] Ruedl C., Storni T., Lechner F., Bächi T., Bachmann M.F. (2002). Cross-presentation of virus-like particles by skin-derived CD8– dendritic cells: A dispensable role for TAP. Eur. J. Immunol..

[62] Ritz U., Seliger B. (2001). The transporter associated with antigen processing (TAP): Structural integrity, expression, function, and its clinical relevance. Mol. Med..

[63] Witkowska-Tobola A.M., Szczerkowska-Dobosz A., Nedoszytko B., Roszkiewicz J. (2004). Polymorphism of the TAP1 gene in Polish patients with psoriasis vulgaris. J. Appl. Genet..

[64] Vašků V., Vašků A., Izakovičová Hollá L., Tschöplová S., Kaňková K., Benáková N., Semrádová V. (2000). Polymorphisms in inflammation genes (angiotensinogen, TAP1 and TNF-β) in psoriasis. Arch. Dermatol. Res..

[65] Ismaïl A., Bousaffara R., Kaziz J., Zili J., El Kamel A., Sfar M.T., Remadi S., Chouchane L. (1997). Polymorphism in transporter antigen peptides gene (TAPI) associated with atopy in Tunisians. J. Allergy Clin. Immunol..

[66] Jadeja S.D., Mansuri M.S., Singh M., Dwivedi M., Laddha N.C., Begum R. (2017). A case-control study on association of proteasome subunit beta 8 (PSMB8) and transporter associated with antigen processing 1 (TAP1) polymorphisms and their transcript levels in vitiligo from Gujarat. PLoS ONE.

[67] Law-Ping-Man S., Toutain F., Rieux-Laucat F., Picard C., Kammerer-Jacquet S., Magérus-Chatinet A., Dupuy A., Adamski H. (2018). Chronic granulomatous skin lesions leading to a diagnosis of TAP1 deficiency syndrome. Pediatr. Dermatol..

[68] Darazam I., Shahrooei M., Hakamifard A., Olyaei N.A., Zerehpoosh E., Gharehbagh F., Hatami F., Lotfollahi L., Mansouri N., Casanova J. (2022). Chronic necrotizing granulomatous skin lesions and MHC class I deficiency syndrome due to TAP2 deficiency. Res. Square..

[69] Kamarashev J., Ferrone S., Seifert B., Boni R., Nestle F., Burg G., Dummer R. (2001). TAP1 down-regulation in primary melanoma lesions: An independent marker of poor prognosis. Int. J. Cancer.

[70] Shoaib Mansuri M., Singh M., Shoab Mansuri M., Jadeja S.D., Gani A.R., Patel R., Dwivedi M., Laddha N.C., Begum R. (2014). Could ER Stress Be a Major Link between Oxidative Stress and Autoimmunity in Vitiligo?. Artic. J. Pigment. Disord..

[71] Glassman S.J. (2011). Vitiligo, reactive oxygen species and T-cells. Clin. Sci..

[72] Linsdell P. (2018). Cystic fibrosis transmembrane conductance regulator (CFTR): Making an ion channel out of an active transporter structure. Channels.

[73] Hanukoglu I., Boggula V.R., Vaknine H., Sharma S., Kleyman T., Hanukoglu A. (2017). Expression of epithelial sodium channel (ENaC) and CFTR in the human epidermis and epidermal appendages. Histochem. Cell Biol..

[74] Engelhardt J.F., Smith S.S., Allen E., Yankaskas J.R., Dawson D.C., Wilson J.M. (1994). Coupled secretion of chloride and mucus in skin of Xenopus laevis: Possible role for CFTR. Am. J. Physiol.—Cell Physiol..

[75] Dong J., Jiang X., Zhang X., Liu K.S., Zhang J., Chen J., Yu M.K., Tsang L.L., Chung Y.W., Wang Y. (2015). Dynamically Regulated CFTR Expression and Its Functional Role in Cutaneous Wound Healing. J. Cell. Physiol..

[76] Larsen E.H., Amstrup J., Willumsen N.J. (2003). β-Adrenergic receptors couple to CFTR chloride channels of intercalated mitochondria-rich cells in the heterocellular toad skin epithelium. Biochim. Biophys. Acta—Biomembr..

[77] Lee A.-Y. (2015). Recent progress in melasma pathogenesis. Pigment Cell Melanoma Res..

[78] Lee A.Y. (2014). An updated review of melasma pathogenesis. Dermatol. Sin..

[79] Hussar D.A., Eckel S.P. (2012). Ivacaftor, vismodegib, and ingenol mebutate. J. Am. Pharm. Assoc..

[80] Burke M.A., Mutharasan R.K., Ardehali H. (2008). The sulfonylurea receptor, an atypical ATP-binding cassette protein, and its regulation of the KATP channel. Circ. Res..

[81] Bryan J., Crane A., Vila-Carriles W., Babenko A., Aguilar-Bryan L. (2005). Insulin Secretagogues, Sulfonylurea Receptors and KATP Channels. Curr. Pharm. Des..

[82] Rafiq M., Flanagan S.E., Patch A.M., Shields B.M., Ellard S., Hattersley A.T., Batra C., Bruining J., Carson D., Codner E. (2008). Effective treatment with oral sulfonylureas in patients with diabetes due to sulfonylurea receptor 1 (SUR1) mutations. Diabetes Care.

[83] Vigneri R., Pezzino V., Wong K.Y., Goldfine I.D. (1982). Comparison of the in Vitro Effect of Biguanides and Sulfonylureas on Insulin Binding to Its Receptors in Target Cells. J. Clin. Endocrinol. Metab..

[84] Vázquez-Sánchez A.Y., Hinojosa L.M., Parraguirre-Martínez S., González A., Morales F., Montalvo G., Vera E., Hernández-Gallegos E., Camacho J. (2018). Expression of KATP channels in human cervical cancer: Potential tools for diagnosis and therapy. Oncol. Lett..

[85] Newfield R.S. (2015). Topical sulfonylurea as a novel therapy for hypertrichosis secondary to diazoxide, and potentially for other conditions with excess hair growth. Med. Hypotheses.

[86] Bednarczyk P., Kicinska A., Laskowski M., Kulawiak B., Kampa R., Walewska A., Krajewska M., Jarmuszkiewicz W., Szewczyk A. (2018). Evidence for a mitochondrial ATP-regulated potassium channel in human dermal fibroblasts. Biochim. Biophys. Acta—Bioenerg..

[87] Gier B., Krippeit-Drews P., Sheiko T., Aguilar-Bryan L., Bryan J., Düfer M., Drews G. (2009). Suppression of KATP channel activity protects murine pancreatic β cells against oxidative stress. J. Clin. Invest..

[88] Bhatti J.S., Sehrawat A., Mishra J., Sidhu I.S., Navik U., Khullar N., Kumar S., Bhatti G.K., Reddy P.H. (2022). Oxidative stress in the pathophysiology of type 2 diabetes and related complications: Current therapeutics strategies and future perspectives. Free Radic. Biol. Med..

[89] Liu Y., Gutterman D.D. (2002). Oxidative Stress and Potassium Channel Function. Clin. Exp. Pharmacol. Physiol..

[90] Robey R.W., To K.K.K., Polgar O., Dohse M., Fetsch P., Dean M., Bates S.E. (2009). ABCG2: A perspective. Adv. Drug Deliv. Rev..

[91] Natarajan K., Xie Y., Baer M.R., Ross D.D. (2012). Role of breast cancer resistance protein (BCRP/ABCG2) in cancer drug resistance. Biochem. Pharmacol..

[92] Ven R., Lindenberg J.J., Reurs A.W., Scheper R.J., Scheffer G.L., Gruijl T.D. (2012). Preferential Langerhans cell differentiation from CD34 ^+^ precursors upon introduction of ABCG2 (BCRP). Immunol. Cell Biol..

[93] Wang P., Sachar M., Lu J., Shehu A.I., Zhu J., Chen J., Liu K., Anderson K.E., Xie W., Gonzalez F.J. (2019). The essential role of the transporter ABCG2 in the pathophysiology of erythropoietic protoporphyria. Sci. Adv..

